# Overexpression of the receptor for advanced glycation end-products in the auditory cortex of rats with noise-induced hearing loss

**DOI:** 10.1186/s12868-021-00642-3

**Published:** 2021-05-21

**Authors:** Chang Ho Lee, Kyung Woon Kim, Da-hye Lee, So Min Lee, So Young Kim

**Affiliations:** Department of Otorhinolaryngology-Head and Neck Surgery, CHA University College of Medicine, 59, Yatap-ro, Bundang-gu, Seongnam, 13496 Gyeonggi-do Korea

**Keywords:** Hearing Loss, Noise-Induced, Brevican, Auditory Cortex, Matrix Metalloproteinase 9

## Abstract

**Background:**

The receptor for advanced glycation end-products (RAGE) is involved in neuroinflammation. This study investigated the changes in RAGE expression following noise-induced hearing loss.

**Methods:**

Three-week-old female SpragueDawley rats were exposed to 115 dB SPL white noise for 4 h daily for 3 d (noise group, n=16). In parallel, age and sex-matched control rats were raised under standard conditions without noise exposure (control group, n=16). After 2 h (noise immediate, n=8) and 4 wk (noise 4-week, n=8) of noise exposure, the auditory cortex was harvested and cytoplasmic and nuclear fractions were isolated. The gene expression levels of tumor necrosis factor alpha (TNF-), interleukin 6 (IL6), interleukin 1 beta (IL1), nuclear factor kappa-light-chain-enhancer of activated B cells (NF-B), and RAGE were evaluated using real-time reverse transcription polymerase chain reaction. The protein expression levels of nuclear RAGE and cytosolic RAGE were evaluated using western blotting. Additionally, matrix metalloproteinase 9 (MMP9) was pharmacologically inhibited in the noise immediate group, and then nuclear and cytosolic RAGE expression levels were evaluated.

**Results:**

The noise immediate and noise 4-week groups exhibited increased auditory thresholds at 4, 8, 16, and 32 kHz frequencies. The genes encoding the pro-inflammatory cytokines TNF-, IL6, IL1, and NF- B were increased 3.74, 1.63, 6.42, and 6.23-fold in the noise immediate group, respectively (P=0.047, 0.043, 0.044, and 0.041). RAGE mRNA expression was elevated 1.42-fold in the noise 4-week group (P=0.032). Cytosolic RAGE expression was increased 1.76 and 6.99-fold in the noise immediate and noise 4-week groups, respectively (P=0.04 and 0.03). Nuclear RAGE expression was comparable between the noise and control groups. matrix metalloproteinase 9 (MMP9) inhibition reduced cytosolic RAGE expression in the noise immediate group (P=0.004).

**Conclusions:**

Noise exposure increased the expression of cytosolic RAGE in the auditory cortex and upregulated pro-inflammatory genes, but this response could be alleviated by MMP9 inhibition.

**Supplementary Information:**

The online version contains supplementary material available at 10.1186/s12868-021-00642-3.

## Background

The receptor for advanced glycation end-products (RAGE) is a transmembrane receptor of the immunoglobulin superfamily and can bind to multiple ligands, including advanced glycation end-products (AGEs) and -amyloid peptide [3, 27]. The AGEs have been observed in aging and neurodegenerative diseases and exert toxicity through RAGE ligation cascades [13, 14]. The AGEs were associated with hearing loss in elderly patients [21]. RAGE ligation activates multiple neuroinflammatory and neurodegenerative pathways [7, 9, 28] and RAGE activation induces proinflammatory cytokines such as TNF-, IL6, IL1 and iNOS in microglia cells [9]. Reportedly, AGEs/RAGE interactions activate nuclear factor kappa-light-chain-enhancer of activated B cells (NF-B) and induce the production of reactive oxygen species (ROS) in the neurodegenerative diseases [28]. Moreover, RAGE mediates -amyloid peptide transport across the bloodbrain barrier via interaction between the -amyloid peptide and RAGE-bearing cells in the vascular wall during the development of cerebral amyloidosis [7]. Although many studies on the central nervous system have examined the role of RAGE in normal aging and disease, the expression and role of these molecules in the context of hearing loss is unclear.

Studies show that noise-induced hearing loss is involved in neuroinflammatory changes in the central auditory pathways [11, 29]. Moreover, chronic noise exposure or hearing loss has been reported to induce neurodegenerative changes and cognitive dysfunctions [18, 23]. To our knowledge, there are no studies on the changes of RAGE expression in the auditory cortex following noise exposure. However, a previous study reported an increase in RAGE expression in rat hippocampus following a 4 wk noise exposure [6]. The authors also described increased expression of pro-inflammatory genes including TNF- and amyloid precursor protein and its cleavage enzymes, - and -secretases [6]. This finding implied that noise exposure induced neuroinflammatory and neurodegenerative changes which might be initiated in the early phase exposure. Similarly, high glucose-induced hearing loss showed increased expression of RAGE, AGE, and NF-B in rat cochlear hair cells [31]. The increased RAGE, AGE, and NF-B levels were attenuated following antioxidant treatment [31], implicating oxidative stress responses in inflammation and RAGE pathways.

We hypothesized that the inflammatory responses after noise exposure were associated with RAGE expression in the auditory cortex. In addition, a persistent change in RAGE expression was anticipated, consistent with previous studies which reported prolonged inflammatory or degenerative effects of noise-induced hearing loss on the central nervous system [18, 22, 23]. To test these hypotheses, we investigated the changes in expression of pro-inflammatory genes and RAGE in the auditory cortex of rats with noise-induced hearing loss after immediate and 4 wk noise exposure. Because a previous study reported the more susceptible changes in the hippocampus to noise exposure than those in the auditory cortex [5], the working memory tests were conducted in the 4 wk noise exposure rats to evaluate the combined hippocampal dysfunction. To explore the mediator of increased cytosolic RAGE expression, a MMP9 inhibitor was administered to rats with noise-induced hearing loss, resulting in the reduction of cytosolic RAGE expression in the auditory cortex.

## Methods

### Animal experiments

The Institutional Animal Care and Use Committee of CHA University Medical School (IACUC190047) approved this study. All animal experiments were conducted in compliance with the guidelines and regulations of the Institutional Animal Care and Use Committee of CHA University Medical School.

**Fig. 1:**
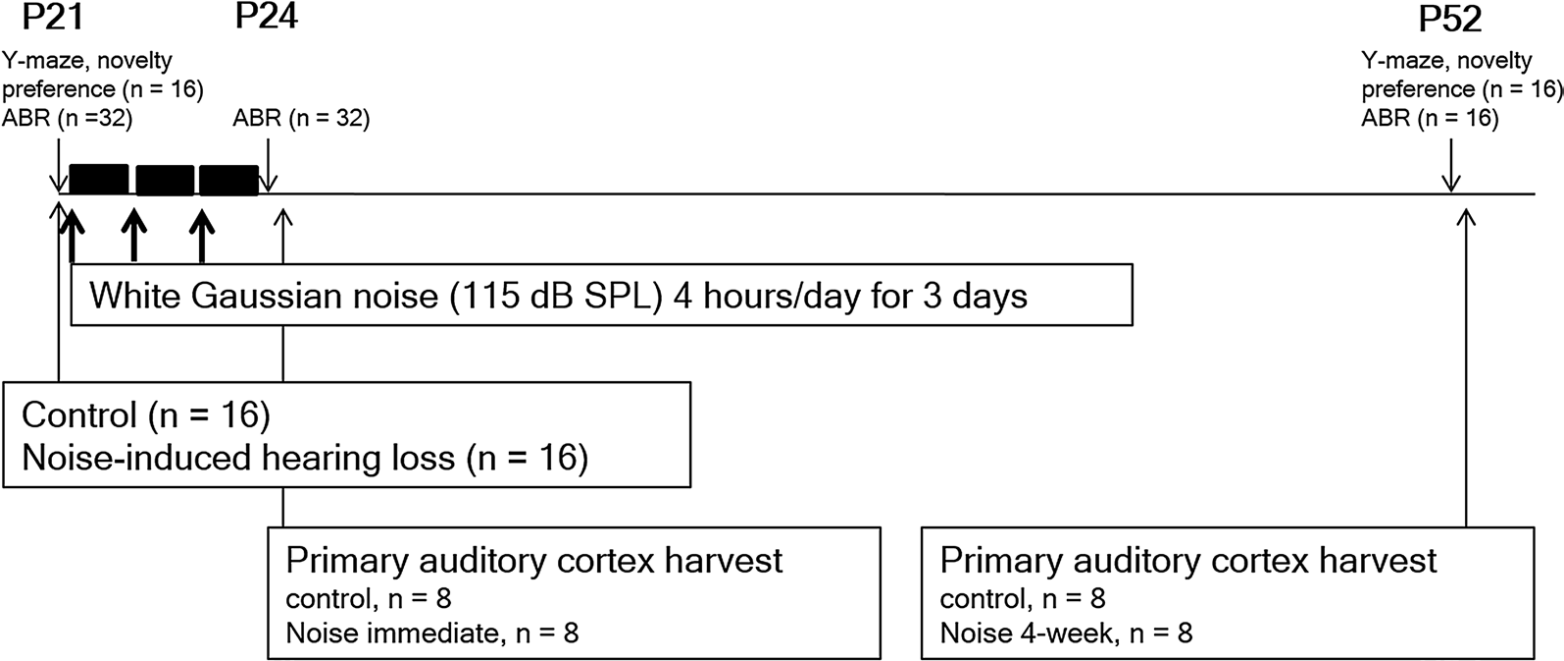
The experimental schedules and measurements of the present study. The white noise (220 kHz, 115 dB SPL), delivered for 4 h per day, for 3 d (noise group, n=16). Immediately and 4 wk after white noise exposure (noise immediate group, n=8 and noise 4-week group, n=8), auditory brainstem responses (ABRs) were measured

Twenty-one-day-old female SpragueDawley rats were exposed to white noise (220 kHz, 115 dB SPL), delivered for 4 h per day, for 3 d (noise group, n=16) (Fig. 1). Free-field electrostatic speaker (Tucker-Davis Technologies, Alachua, FL, USA) was placed on top of the chamber to deliver noise. Rats were exposed to noise in awakening state. The control group was raised under standard conditions with about 4060 dB SPL background noise (n=16). Immediately and 4 wk after white noise exposure, auditory brainstem responses (ABRs) were measured in all rats (SmartEP; Intelligent Hearing System, Miami, FL, USA). Needle electrodes were inserted into the vertex and behind the ipsilateral pinna and plastic earphones were plugged into external auditory canals. An EC1 electrostatic speaker delivered 4, 8, 16, and 32 kHz of tone-burst stimuli (duration, 1562 s; envelope, Blackman; stimulation rate, 21.1/s; low pass filter 5000 Hz; high pass filter, 0 Hz). Amplified evoked responses with 1,024 sweeps were averaged. From 90 dB SPL, the tone-burst stimuli were decreased at 10 dB SPL intervals. The lowest sound intensity that evoked wave II was defined as the auditory threshold because wave II was the most prominent waveform of rat ABRs [1, 4, 26].

To evaluate the effect of MMP9 inhibition on RAGE expression following noise exposure, MMP9 inhibitor (SB-3CT, 25 mg/kg) was intraperitoneally injected before noise exposure (noise+SB-3CT group, n=5). The protein expression levels of nuclear and cytosolic RAGE and MMP9 in the noise+SB-3CT group was compared with those of control and noise groups (each n=5).

### Spatial working memory in the Y-maze

The Y-maze test for spatial working memory was conducted 4 wk after noise exposure in the noise 4-week and control groups as previously described (n = 8 for each group) [16]. Briefly, the black plastic Y-maze consisted of three identical arms (11 cm wide, 50 cm long, 30 cm high, 120 apart). Rats were placed at the distal end of start arm and allowed to explore the maze freely for 5 minutes. The number of entries into each arm were recorded. The number of total arm entry and spontaneous alternation percentage (%) were compared between noise 4-week group and control group (each n = 8). Entries were counted when the rats passed over the midpoint of the arm. Entries into new arms were considered successful alternations, as opposed to entering the two previously visited arms. Alternation percentage (%) was calculated using the formula: (Number of successful alternations/[Total arm entries-2]) 100.

### Novelty preference tests

A Y-maze identical to the one used for spatial working memory tests was used for the novelty preference tests, which were conducted 2-3 d after the spatial working memory tests in the noise 4-week and control groups (n = 8 for each group). In the exposure phase, rats were placed in the two arms of start arm and familiar arm in the first 5-minutes but the third arm, the novel arm, was closed by the black block. After exposure phase, rats were placed in a standard cage for 5-minutes. Then in the test phase, rats were placed back in the start arm and allowed to explore all three arms for 5-minutes by removing the black block. The novelty preference (%) were compared between noise 4-week group and control group (each n = 8). The novelty preference (%) was assessed using the formula: (number of novel arm entries / total arm entries) 100.

After the tests, the rats were sacrificed using carbon dioxide inhalation as previously described [15]. The flow velocity of inhaled gas was 5-6L/min and the rats were placed in 25.40 cm * 48.26 cm * 22.86 cm size cage. The auditory cortex was micropunched and rapidly frozen at 20 C (n=8 for each group). Paxinos and Watson coordinates (A/P= 2.7~5.8 mm, M/L=6.4~8.7 mm) were used to localize the auditory cortex [24]. Both sides of auditory cortex were harvested and they were combined together for analyses. Four rats per group were analyzed for each experiment (quantitative real-time reverse transcription polymerase chain reaction [RT-PCR] and western blot). For 3B-SCT study, three rats per group were analyzed for western blotting and two rats per group were analyzed for immunofluorescence study.

### mRNA expression levels of pro-inflammatory genes

The expression levels of TNF-, IL6, IL1, NF-B, and RAGE mRNA in the auditory cortex were analyzed using quantitative real-time reverse transcription polymerase chain reaction (RT-PCR). Total RNA from each brain tissue was extracted using the TRI Reagent (SigmaAldrich, St. Louis, MO, USA). The purified RNA was checked for purity and quantity by measuring the 260/280 nm absorbance ratio on a NanoDrop^TM^1000 spectrophotometer (Thermo Scientific, Madison, WI, USA). Only samples with values greater than 1.8 for the 260/280 ratio and greater than 1.5 for the 260/230 ratio were eligible for RT-PCR. The quality of the RNA was checked using an Agilent 2100 Bioanalyzer (Agilent Technologies, Santa Clara, CA, USA) ('Genomics Agilent') ('Genomics Agilent') ('Genomics Agilent') ^22^ ('Genomics Agilent') ('Genomics Agilent') ('Genomics Agilent') ('Genomics Agilent') ('Genomics Agilent') ('Genomics Agilent') ('Genomics Agilent') [12]. Only samples with a value greater than 7.0 for the RNA Integrity Number were eligible for RT-PCR. No samples were excluded due to low RNA quality. Forward and reverse oligonucleotides used for reverse transcription are listed on Table 1. The target gene mRNA expression levels were expressed as a percentage of the expression level of glyceraldehyde 3-phosphate dehydrogenase mRNA.

**Table 1:** Oligonucleotide primer sequences for quantitative reverse transcriptase polymerase chain reaction

Gene	Primer sequence (forward)	Primer sequence (reverse)	Annealing temperature (C)	Product size (bp)	RefSeq Number
*TNF*	5'- CGTCAGCCGATTTGCCATTT -3'	5'- TCCCTCAGGGGTGTCCTTAG -3'	60	88	NM_012675.3
*IL6*	5'- AGAGACTTCCAGCCAGTTGC -3'	5'- TGAAGTCTCCTCTCCGGACT -3'	60	88	NM_012589.2
*IL1*	5'- CACCTTCTTTTCCTTCATCTTTG -3'	5'- GTCGTTGCTTGTCTCTCCTTGTA -3'	60	241	NM_031512.2
*NFB*	5'- TGTCTGCACCTGTTCCAAAGA -3'	5'- TGCCAGGTCTGTGAACACTC-3'	60	143	NM_199267.2
*RAGE*	5'- GGAAGGACTGAAGCTTGGAAGG -3'	5'- TCCGATAGCTGGAAGGAGGAGT -3'	60	102	NM_053336.2
*GAPDH*	5'- ATTGTTGCCATCAACGACCC -3'	5'- TGACTGTGCCGTTGAACTTG -3'	60	94	NM_017008.4

### Isolation of nuclear and cytoplasmic extract

To estimate the expression levels of nuclear and cytosolic RAGE, nuclear and cytoplasmic fractions were extracted using an NE-PER Nuclear Cytoplasmic Extraction Reagent kit (Pierce, Rockford, IL, USA) according to the manufacturer's instructions. Briefly, the temporal cortex tissue was washed twice with cold PBS and centrifuged at 500*g* for 5 min. The pellet was resuspended in 400L of cytoplasmic extraction reagent I by homogenizing. The suspension was then incubated on ice for 10 min followed by the addition of 22L of cytoplasmic extraction reagent II. The mixture was vortexed for 5 s, incubated on ice for 1 min and centrifuged for 5 min at 16000*g*. The supernatant (cytoplasmic extract) was transferred to a microcentrifuge tube. The insoluble pellet was resuspended in 200L of nuclear extraction reagent by vortexing for 15 s, incubated on ice for 10 min, and centrifuged for 10 min at 16000*g*. The resulting supernatant constituted the nuclear extract.

### Protein expression levels of Intra- and extra-RAGE and MMP9

Protein expression levels were analyzed using western blotting. The brain tissue was lysed using the NE-PER Nuclear Cytoplasmic Extraction Reagent kit (Pierce, Rockford, IL, USA) and radioimmunoprecipitation assay buffer (Cell Signaling Technology, Danvers, MA, USA). Protein concentration was measured using the Bio-Rad Protein Assay Kit. The proteins were separated using 8% sodium dodecyl sulfatepolyacrylamide gel electrophoresis and transferred to polyvinylidene difluoride membranes (Merck Millipore, Burlington, MA, USA). The membranes were soaked in blocking buffer (5% nonfat dry milk in Tris-buffered saline containing Tween-20 [TBS-T]) for 1 h and incubated with primary antibodies against intra (nuclear) RAGE (ab3611, Rabbit polyclonal, Abcam), extra (cytosolic) RAGE (MAB1179, Rat monoclonal, R&D system), MMP9 (Rabbit monoclonal, abcam, UK), and -actin (D6A8, rabbit mAb; Cell Signaling Technology). Horseradish peroxidase (HRP)-conjugated secondary antibodies (anti-rabbit IgG, HRP-linked antibody; Cell Signaling Technology, #7074S and anti-Rat IgG, HRP-conjugated Antibody; R&D system, #HAF005) were used to detect the immunoreactive proteins, and the samples were visualized using an enhanced chemiluminescence kit (Bio-Rad). Protein expression levels were calculated using ImageJ gel analysis software (National Institutes of Health, Bethesda, MD, USA). The protein levels are expressed as a percentage of the -actin level.

### Immunofluorescence

The expressions of MMP9 and RAGE (cytosol) in the auditory cortex were analyzed using immunofluorescence studies. Paraffin sections of brain tissue were prepared with 10- m thickness. The brain sections were mounted on glass slides and deparaffinized in xylene for 10 min. Then the slides were repeatedly washed in ethanol (199%, 75%, and 50%) and three times in PBS for 5 min. The blocking was performed in 10% donkey blocking serum (Vector Labs, Burlingame, CA, USA) for 1 h followed by the incubation with the primary antibodies (extra [cytosolic] RAGE [MAB1179, Rat monoclonal, R&D system], MMP9 [Rabbit monoclonal, abcam, UK]) overnight at 4 C. The slides were washed three times in PBS for 10 min. The secondary antibodies (anti-rat and anti-rabbit Alexa 594) were incubated for 2 h. The slides were incubated with 4,6-diamidino-2-phenylindole (DAPI) (SigmaAldrich, St. Louis, MO, USA) for 5 min. The slides were washed three times in PBS for 10 min. Then the slides were covered. The auditory cortex in Paxinos and Watson coordinates (A/P=-2.7~5.8 mm, M/L=6.4~8.7 mm, V/D=2.9~4.5 mm) were evaluated using TCS SP5II confocal microscope (Leica, Wetzlar, Germany) [25].

### Statistical analysis

The differences in gene expression, Y-maze, and novelty preference tests between the noise and control groups were analyzed with the MannWhitney U test. Paired t-test was used to analyze the differences of ABR thresholds between pre- and post-noise exposures. Unpaired t-test was used to analyze the differences of ABR thresholds between control and noise groups. Normality tests were conducted using KolmogorovSmirnov and ShapiroWilk tests. Statistical significance was set at P<0.05. SPSS 21.0 (IBM Corp., Armonk, NY, USA) was used for statistical analysis. All graphs were presented with meanstandard error (SE).

## Results

### ABR threshold shifts after noise exposures

**Fig. 2:**
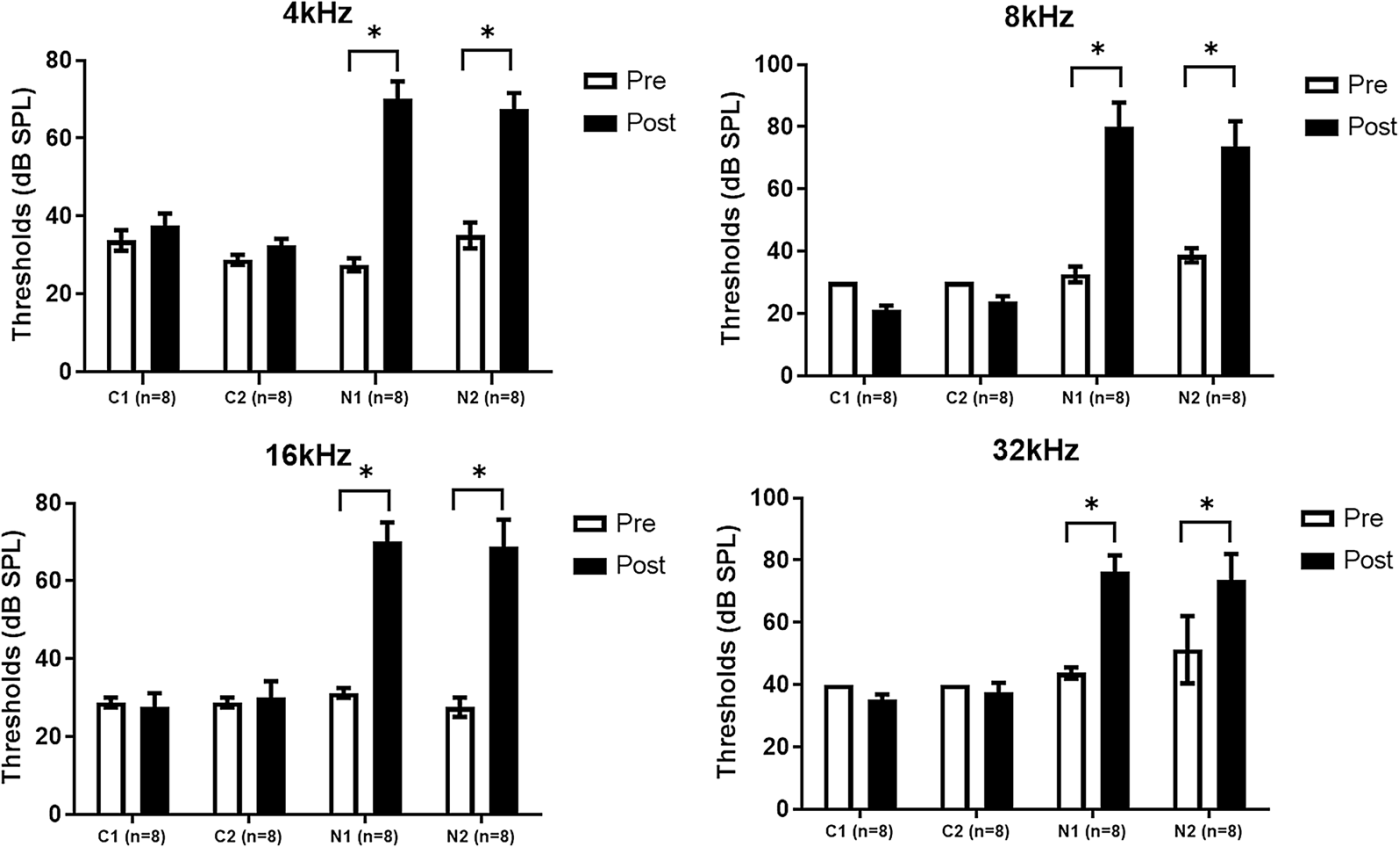
The auditory brainstem response (ABR) thresholds at pre- and post-noise exposures. ABR thresholds were increased after noise exposure, compared to pre-noise exposure (*P<0.05, paired t-test, error bar=standard errors)

Average ABR thresholds were not significantly different between control and noise groups before noise exposures (Fig. 2 and Additional file 1: Table S1). Following noise exposures, the ABR thresholds increased and persisted for 4 wk post-exposure.

### Spatial activity changes after noise exposure

**Fig. 3:**
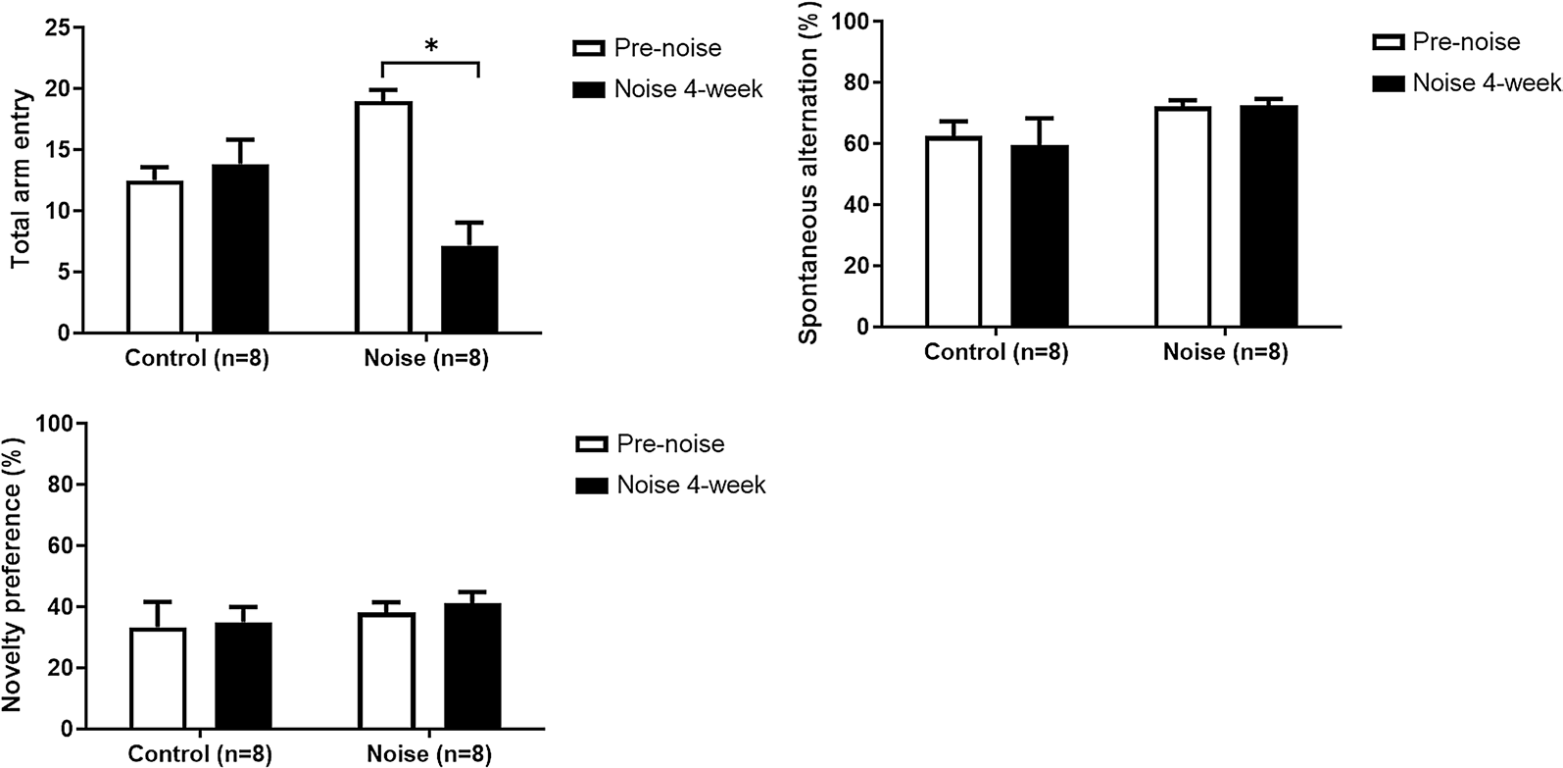
Y-maze and novelty preference tests at 4 wk after white noise exposure. The number of total arm entry was decreased in the noise 4-week group, compared to control group (7.17 vs. 13.83, P=0.036). The proportions of spontaneous alternation and novelty preference were similar between control and noise 4-week groups. (error bar=standard errors)

In the Y-maze tests, the number of total arm entry was decreased in the noise 4-week group, compared to control group (7.17 vs. 13.83, P=0.036) (Fig. 3). However, the proportions of spontaneous alternation were similar between control and noise 4-week groups (59.56 vs. 72.73%, P=0.087). The proportions of novelty preference were also comparable between control and noise 4-week groups (35.00 vs. 41.23%, P=0.41).

### Changes of pro-inflammatory gene expressions in noise-induced hearing loss rats

**Fig. 4:**
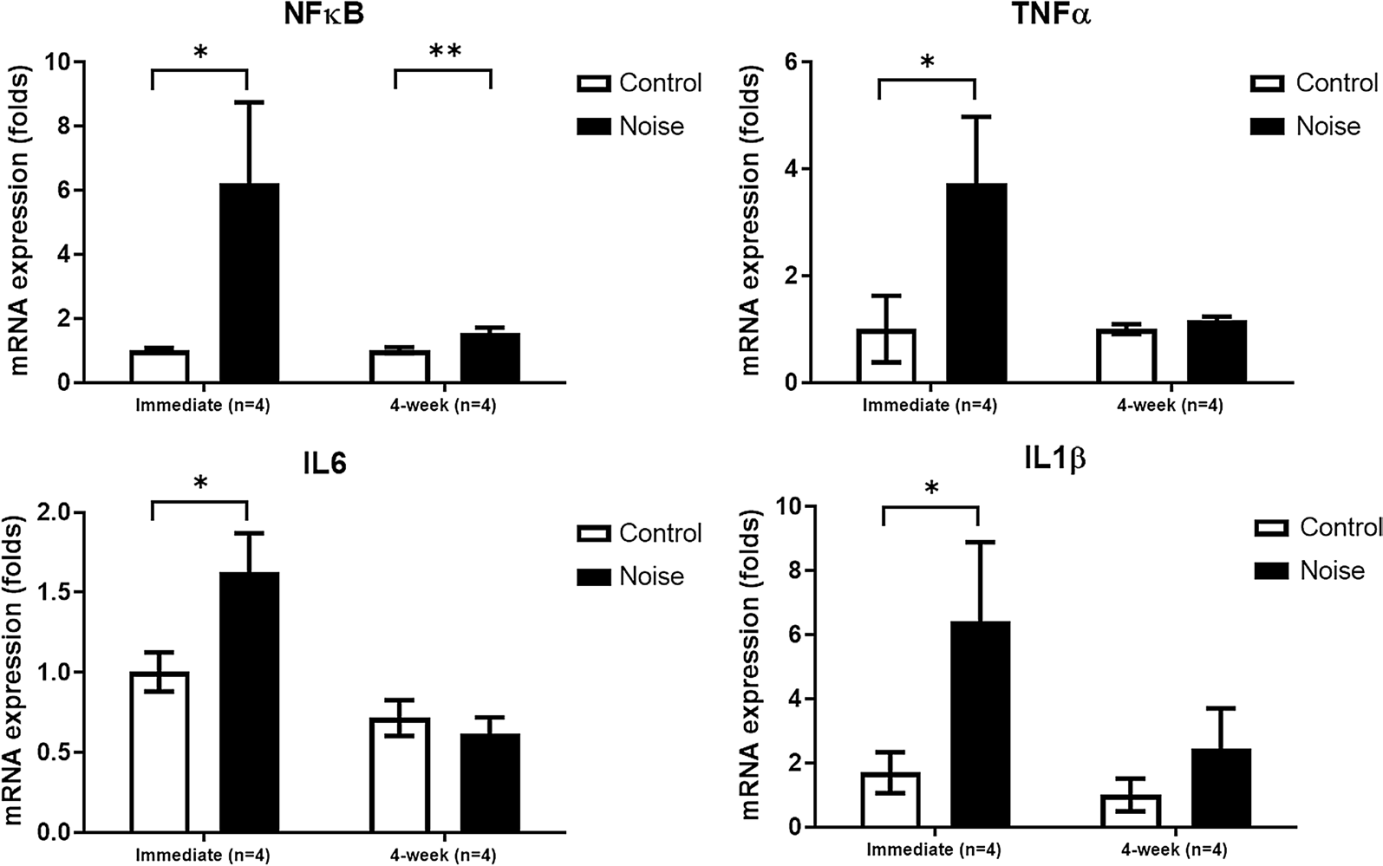
The noise immediate group demonstrated increased mRNA expression levels of tumor necrosis factor alpha (TNF-), interleukin 6 (IL6), interleukin 1 beta (IL1), and nuclear factor kappa-light-chain-enhancer of activated B cells (NF-B), compared to control group (*P<0.05, MannWhitney U test). The noise 4-week group showed lower mRNA expression level of NF-B than control group (**P<0.05, MannWhitney U test). (error bar=standard errors)

The mRNA expression levels of pro-inflammatory genes were increased in the noise immediate group compared to the control group (Fig. 4). TNF-, IL6, IL1, and NF-B mRNA expression levels were elevated 3.74, 1.63, 6.42, and 6.23-fold in the noise immediate group than in the control group (P=0.047, 0.043, 0.044, and 0.041). In the noise 4-week group, NF-B mRNA expression level was 1.55-fold higher than that in the control group (P=0.016). However, TNF-, IL6, and IL1 mRNA expression levels were not significantly different between the noise 4-week and control groups (P=0.162, 0.527, and 0.313).

### RAGE expressions in noise-induced hearing loss rats

**Fig. 5:**
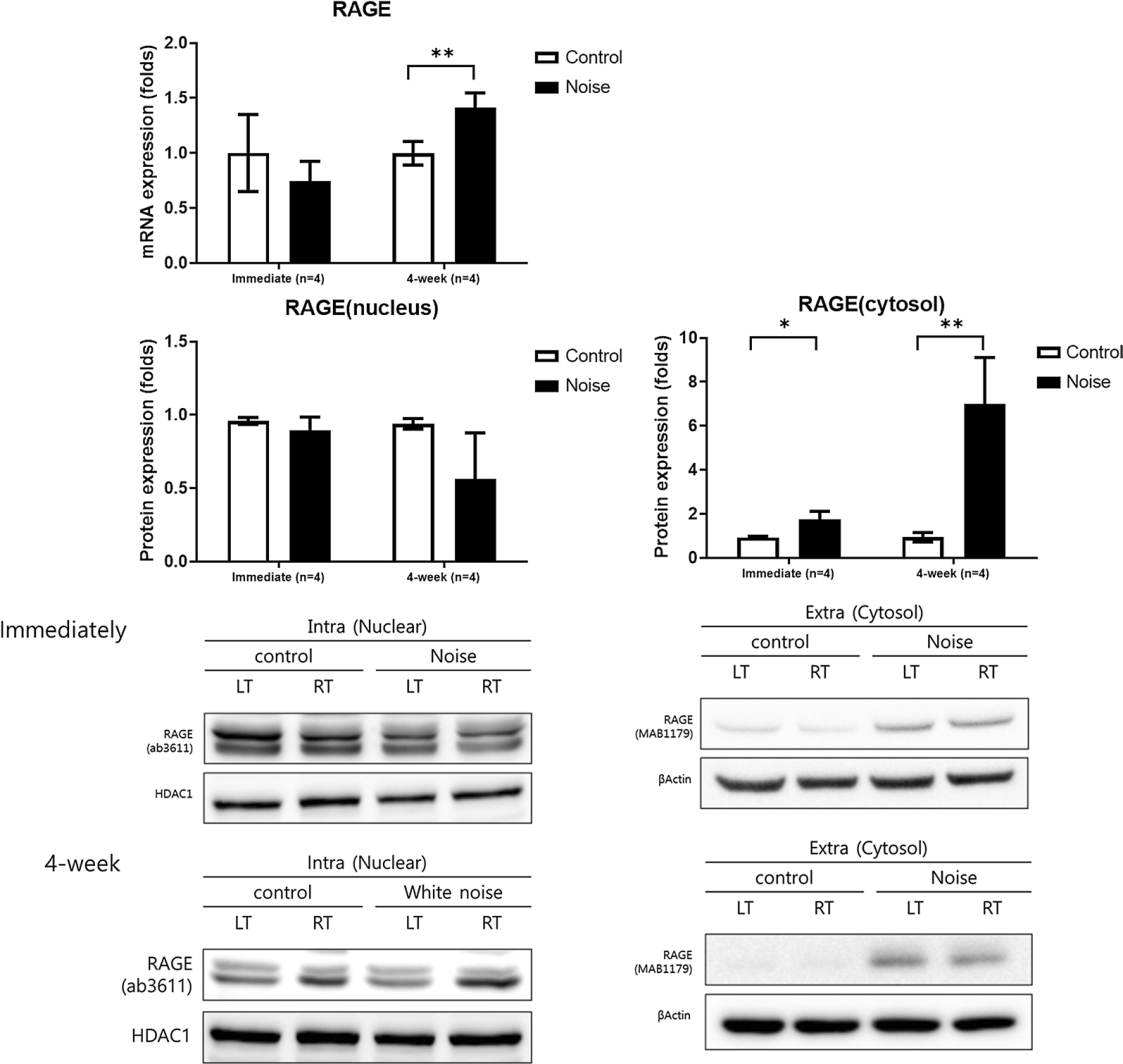
The noise immediate and noise 4-week groups demonstrated increased protein expression level of cytosolic receptor for advanced glycation end-products (RAGE) compared to their control groups (*P<0.05 between noise immediate vs. control groups and **P<0.05 between noise 4-week vs. control groups, MannWhitney U test). Nuclear RAGE expression was comparable between the noise and control groups. (error bar=standard errors)

RAGE mRNA expression was elevated 1.42-fold in the noise 4-week group (P=0.032). The protein expression level of cytosolic RAGE was increased in both noise immediate and noise 4-week groups, compared to control groups (Fig. 5, Additional file 2: Figure S1). The noise immediate group showed 1.76-fold higher expression of cytosolic RAGE than did the control group (P=0.04). The noise 4-week group showed 6.99-fold higher expression of cytosolic RAGE than did the control group (P=0.029). In contrast, the protein expression level of nuclear RAGE was not significantly different between the noise and control groups (P=0.509 and P=0.279, respectively).

### RAGE suppression by MMP9 inhibition in noise-induced hearing loss rats

**Fig. 6:**
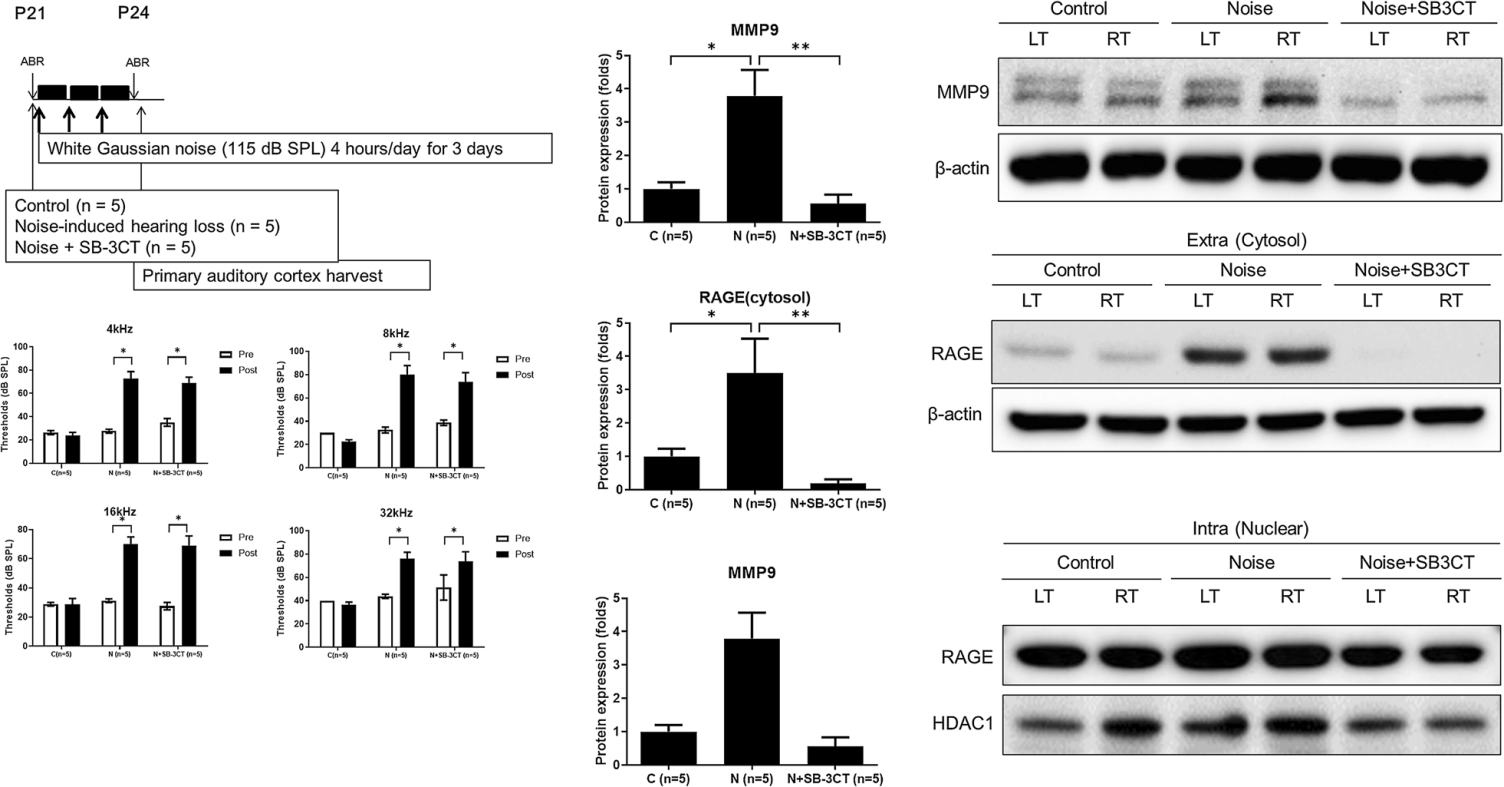
The administration of MMP9 inhibitor (SB-3CT) reduced the cytosolic RAGE expressions in noise+SB-3CT group, compared to noise group (*P<0.05 between noise vs. control groups and **P<0.05 between noise vs. noise+SB-3CT groups, MannWhitney U test). (error bar=standard errors)

**Fig. 7:**
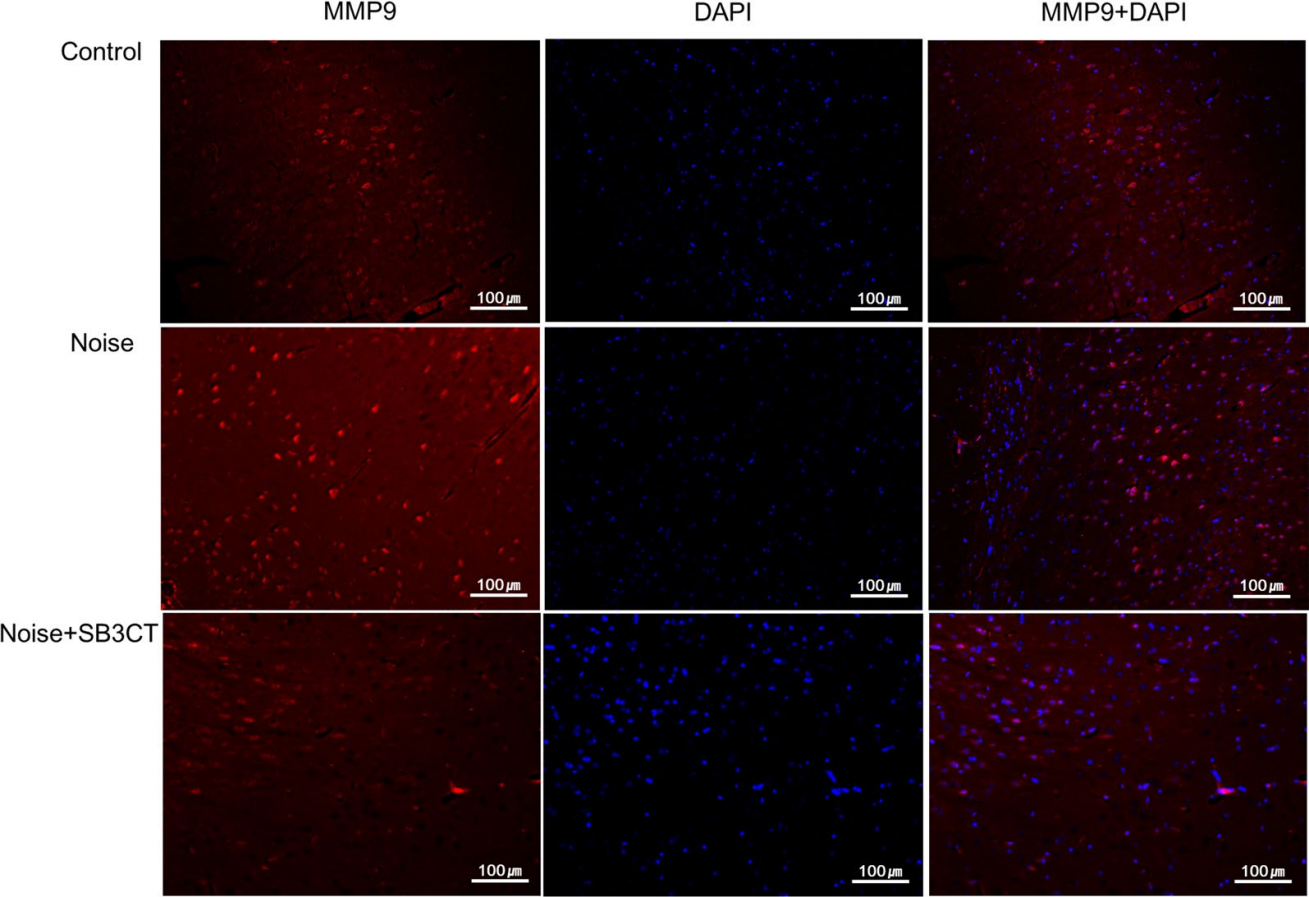
The matrix metalloproteinase 9 (MMP9) expression level (red) in the auditory cortex was increased in the noise group. On the other hands, the MMP9 expression level in the auditory cortex was decreased in the noise+SB-3CT group. (blue: DAPI)

**Fig. 8:**
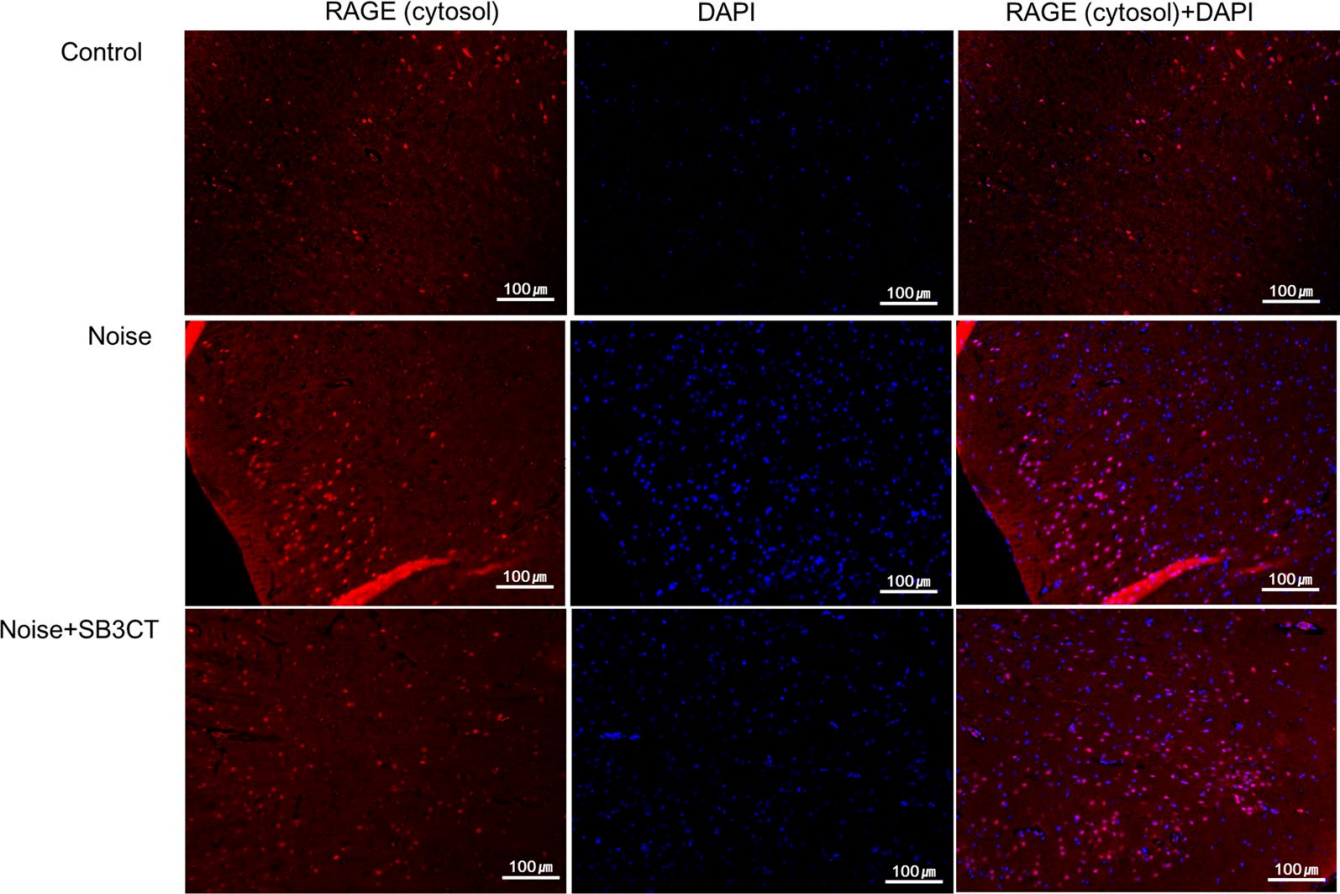
The cytosolic receptor for advanced glycation end-products (RAGE) expression level (red) in the auditory cortex was increased in the noise group. On the other hands, the cytosolic RAGE expression level in the auditory cortex was decreased in the noise+SB-3CT group. (blue: DAPI)

To delineate the molecular mediators for RAGE increase in the noise group, a MMP9 inhibitor (SB-3CT) was administered (Fig. 6, Additional file 2: Figure S2). Average ABR thresholds were not significantly different between noise and noise+SB-3CT groups after noise exposures (Additional file 3: Table S2). In the noise+SB-3CT group, the expression levels of MMP9 and cytosolic RAGE were both reduced compared to those in the noise group. MMP9 expression level was 0.56 (SE=0.27) and 3.79 (SE=0.78) in noise+SB-3CT and noise groups, respectively (P=0.004). The expression level of cytosolic RAGE was 0.19 (SE=0.12) and 3.50 (SE=1.03) in noise+SB-3CT and noise groups, respectively (P<0.001). In contrast, the expression level of nuclear RAGE was similar between noise and noise+SB-3CT groups (0.59 vs. 1.29, P=0.11). The expression levels of MMP9 and cytosolic RAGE were increased in the auditory cortex of noise group compared to control group in the immunofluorescence studies (Figs. 7, 8). The noise+SB-3CT group showed decreased expression levels of MMP9 and cytosolic RAGE compared to noise group (Figs. 7, 8).

## Discussion

Noise-induced hearing loss increased the expression of genes encoding pro-inflammatory cytokines TNF-, IL6, IL1, and NF-B in the auditory cortex, immediately after noise exposure. The increase in the expression of these pro-inflammatory genes was attenuated at 4 wk after noise exposure. However, the expression of cytosolic RAGE steadily increased until 4 wk after noise exposure. The inhibition of MMP9 alleviated the increase of cytosolic RAGE expression. The findings of the present study demonstrate immediate pro-inflammatory impacts and sustained RAGE increment in the auditory cortex following noise exposure.

Several recent studies have described cognitive deficits following noise exposures [18, 22, 23]. Together with age-related and long-term hearing loss models, young adult CBA/CAJ mice (1.52 mo old) showed decreased spatial learning and memory as early as 3 mo after noise exposure [18]. Moreover, a previous research demonstrated earlier changes of oxidative stress and tau phosphorylation in the hippocampus to the 1 week of noise exposure, while the auditory cortex did not show significant changes until the 3 weeks of noise exposure [5]. In this study, only the total number of arm entry in the Y-maze test was decreased and spontaneous alternation and novelty preference were not decreased in the noise 4-week group compared to control group. Although the cognitive deficit was not definite, this study demonstrated the changes in the expression of neuroinflammatory genes in this early period after noise exposure.

The study showed that the expression of pro-inflammatory genes was increased immediately after noise exposure. In line with these results, prior studies reported that noise-induced hearing loss cause inflammatory responses, including the activation of microglia and the release of proinflammatory cytokines in the auditory cortex and cochlear nucleus [2, 11, 29]. TNF- expression was elevated immediately (12 h) after noise exposure although the increment was attenuated over time [29]. It was also shown that TNF- played a crucial role in neuroinflammation in the auditory cortex following noise exposure. Accordingly, the inhibition of TNF- in a genetic knockout or by a pharmacological inhibitor decreased the expression of the neuroinflammatory genes TNF-, IL1, IL18, and Nod-like receptor protein 3, and prevented microglial activation and tinnitus in mice with noise-induced hearing loss[29].

This study also showed that the expression of cytosolic RAGE was increased immediately after noise exposure. In addition, the increased RAGE level was sustained at 4 wk after noise exposure. In contrast, the elevated expression levels of the pro-inflammatory genes were attenuated at 4 wk. Interestingly, NF-B expression was higher in the noise 4-week group than in the control group possibly due to long-term effects of noise-induced hearing loss on the central nervous system. Although little is known about the changes in the auditory cortex, it has been suggested that persistent neurodegenerative changes in the hippocampus which progressed after transient elevation of oxidative stress and inflammation occurred after noise exposures [18, 22]. A previous study demonstrated neurodegeneration in the hippocampus and other cognitive deficits in mice at 3 mo after noise exposure, and elevated oxidative stress molecules including corticosterone, superoxide dismutase, malondialdehyde adduct, and reactive oxygen species, which were transiently increased after noise exposure and then normalized [18]. In another study, p-tau and lipofuscin were increased in the mice hippocampus at 12 mo after noise exposure [22]. In an aminoglycoside-induced hearing loss, RAGE expression was increased mostly in cochlea epithelial cells, while TNF-, IL1 or IL10 expression was not detected in the cochlear perilymph [17]. The authors suggested an increase in extracellular high mobility group box 1, a known RAGE ligand, might regulate the epithelial reorganization of the injured organ of Corti [17]. Therefore, the sustained RAGE expression observed in this study might influence the remodeling of and degenerative changes in the auditory cortex. Indeed, a clinical study presented high level of AGEs in hearing loss patients [21].

MMP9 expression was increased in the noise-immediate group. However, inhibition by SB-3CT reduced MMP9 and cytosolic RAGE expression. A previous study reported increased MMP9-induced shedding and activation of RAGE, which mediated neuroinflammation and the reduction of perineuronal nets (PNNs) in the anterior cingulate cortex in schizophrenia mice models [10]. Likewise, increased MMP9 expression and decreased PNNs in the auditory cortex following hearing loss have also been reported [8, 20]. Moreover, MMP9 expression was increased in the auditory cortex and hippocampus in the age-related hearing loss model, which demonstrated cognitive decline [8]. The attenuation of PNNs density in mice auditory cortex lasted at least 30 d following noise exposure, suggesting a link with the increased excitability of cortex and tinnitus [20]. The relationship between MMP9 and PNNs expression has been suggested in previous studies [10, 19, 30]. In a Fmr1 knock-out mice, the expression of PNNs in the auditory cortex was reduced, but was normalized and the auditory hypersensitivity relieved following the genetic knockdown of MMP9 [19, 30]. Thus, the increase in MMP9 could be one mediator of RAGE activation and neuroinflammation in the auditory cortex in the noise-induced hearing loss model used in this study. The decrease in RAGE expression in the MMP9 inhibitor administered mice (noise+SB-3CT) supports this hypothesis.

## Conclusion

The immediate noise exposure increased the expression of pro-inflammatory genes and cytosolic RAGE in the auditory cortex. RAGE expression was further increased with a sustained 4 wk noise-induced hearing loss. The inhibition of MMP9 was accompanied the decreased cytosolic RAGE expression following noise exposure. In conclusion, the inflammation responses in the auditory cortex following noise exposure were initiated as immediately as within 24 h. Although the acute inflammatory cytokines were normalized, the sustained damages in the auditory cortex were sustained until 4-weeks after noise exposure. The increased levels of MMP9 and cytosolic RAGE were presumed to be involved in these chronic inflammatory responses in the auditory cortex.

## Supplementary Information

**Additional file 1**: **Table S1**. The auditory brainstem response (ABR) thresholds at pre- and post-noise exposures.

**Additional file 2**: **Figure S1**. The gel images for Figure 5. **Figure S2**. The gel images for figure 6.

**Additional file 3**: **Table S2**. The auditory brainstem response (ABR) thresholds at pre- and post-noise exposures in control, noise, and noise+SB-3CT groups.

## Data Availability

The data used in our study are available from the authors on reasonable request.

## Abbreviations

RAGE: Advanced glycation end-products
AGEs: Advanced glycation end-products
TNF-: Tumor necrosis factor alpha
IL6: Interleukin 6
IL1: Interleukin 1 beta
NF-B: Nuclear factor kappa-light-chain-enhancer of activated B cells
ROS: Reactive oxygen species
MMP9: Matrix metalloproteinase 9
ABRs: Auditory brainstem responses
RT-PCR: Quantitative real-time reverse transcription polymerase chain reaction
DAPI: 4,6-Diamidino-2-phenylindole
PNNs: Perineuronal nets

## References

[1] Alvarado JC, Fuentes-Santamaria V, Jareno-Flores T, Blanco JL, Juiz JM (2012). Normal variations in the morphology of auditory brainstem response (ABR) waveforms: a study in Wistar rats. Neurosci Res.

[2] Baizer JS, Wong KM, Manohar S, Hayes SH, Ding D, Dingman R, Salvi RJ (2015). Effects of acoustic trauma on the auditory system of the rat: The role of microglia. Neuroscience.

[3] Cai Z, Liu N, Wang C, Qin B, Zhou Y, Xiao M, Chang L, Yan LJ, Zhao B (2016). Role of RAGE in Alzheimer's Disease. Cell Mol Neurobiol.

[4] Chang A, Li C, Huang J, Pan W, Tian Y, Tang J (2018). Auditory brainstem response and outer hair cell whole-cell patch clamp recording in postnatal rats. J Vis Exp.

[5] Cheng L, Wang SH, Huang Y, Liao XM (2016). The hippocampus may be more susceptible to environmental noise than the auditory cortex. Hear Res.

[6] Cui B, Li K, Gai Z, She X, Zhang N, Xu C, Chen X, An G, Ma Q, Wang R (2015). Chronic noise exposure acts cumulatively to exacerbate Alzheimer's disease-like amyloid-beta pathology and neuroinflammation in the rat hippocampus. Sci Rep.

[7] Deane R, Du Yan S, Submamaryan RK, LaRue B, Jovanovic S, Hogg E, Welch D, Manness L, Lin C, Yu J, Zhu H, Ghiso J, Frangione B, Stern A, Schmidt AM, Armstrong DL, Arnold B, Liliensiek B, Nawroth P, Hofman F, Kindy M, Stern D, Zlokovic B (2003). RAGE mediates amyloid-beta peptide transport across the blood-brain barrier and accumulation in brain. Nat Med.

[8] Dong Y, Guo CR, Chen D, Chen SM, Peng Y, Song H, Shi JR (2018). Association between agerelated hearing loss and cognitive decline in C57BL/6J mice. Mol Med Rep.

[9] Dukic-Stefanovic S, Gasic-Milenkovic J, Deuther-Conrad W, Munch G (2003). Signal transduction pathways in mouse microglia N-11 cells activated by advanced glycation endproducts (AGEs). J Neurochem.

[10] Dwir D, Giangreco B, Xin L, Tenenbaum L, Cabungcal JH, Steullet P, Goupil A, Cleusix M, Jenni R, Chtarto A, Baumann PS, Klauser P, Conus P, Tirouvanziam R, Cuenod M, Do KQ (2019). MMP9/RAGE pathway overactivation mediates redox dysregulation and neuroinflammation, leading to inhibitory/excitatory imbalance: a reverse translation study in schizophrenia patients. Mol Psychiatry..

[11] Fuentes-Santamaria V, Alvarado JC, Melgar-Rojas P, Gabaldon-Ull MC, Miller JM, Juiz JM (2017). The role of glia in the peripheral and central auditory system following noise overexposure: contribution of TNF-alpha and IL-1beta to the pathogenesis of hearing loss. Front Neuroanat.

[12] Genomics Agilent. https://www.agilent.com/en/products/genomics-agilent.

[13] Glenn JV, Stitt AW (2009). The role of advanced glycation end products in retinal ageing and disease. Biochim Biophys Acta.

[14] Grillo MA, Colombatto S (2008). Advanced glycation end-products (AGEs): involvement in aging and in neurodegenerative diseases. Amino Acids.

[15] Guidelines for euthanasia of rodents using carbon dioxide. https://oacu.oir.nih.gov/sites/default/files/uploads/arac-guidelines/b5_euthanasia_of_rodents_using_carbon_dioxide.pdf.

[16] Kim SY, Lee DH, Park S, Kim BG, Jang AS, Oh SH, Lee JH, Suh MW, Park MK (2019). Neuronal and perineuronal changes of cerebral cortex after exposure to inhaled particulate matter. Sci Rep.

[17] Ladrech S, Mathieu M, Puel JL, Lenoir M (2013). Supporting cells regulate the remodelling of aminoglycoside-injured organ of Corti, through the release of high mobility group box 1. Eur J Neurosci.

[18] Liu L, Shen P, He T, Chang Y, Shi L, Tao S, Li X, Xun Q, Guo X, Yu Z, Wang J (2016). Noise induced hearing loss impairs spatial learning/memory and hippocampal neurogenesis in mice. Sci Rep.

[19] Lovelace JW, Wen TH, Reinhard S, Hsu MS, Sidhu H, Ethell IM, Binder DK, Razak KA (2016). Matrix metalloproteinase-9 deletion rescues auditory evoked potential habituation deficit in a mouse model of Fragile X Syndrome. Neurobiol Dis.

[20] Nguyen A, Khaleel HM, Razak KA (2017). Effects of noise-induced hearing loss on parvalbumin and perineuronal net expression in the mouse primary auditory cortex. Hear Res.

[21] Niihata K, Takahashi S, Kurita N, Yajima N, Omae K, Fukuma S, Okano T, Nomoto Y, Omori K, Fukuhara S, Group Sukagawa Study (2018). Association between accumulation of advanced glycation end-products and hearing impairment in community-dwelling older people: a cross-sectional Sukagawa study. J Am Med Dir Assoc.

[22] Park SY, Kim MJ, Kim HL, Kim DK, Yeo SW, Park SN (2018). Cognitive decline and increased hippocampal p-tau expression in mice with hearing loss. Behav Brain Res.

[23] Park SY, Kim MJ, Sikandaner H, Kim DK, Yeo SW, Park SN (2016). A causal relationship between hearing loss and cognitive impairment. Acta Otolaryngol.

[24] Paxinos G, Watson C (2005). The rat brain in stereotaxic coordinates.

[25] Polley DB, Read HL, Storace DA, Merzenich MM (2007). Multiparametric auditory receptive field organization across five cortical fields in the albino rat. J Neurophysiol.

[26] Scimemi P, Santarelli R, Selmo A, Mammano F (2014). Auditory brainstem responses to clicks and tone bursts in C57 BL/6J mice. Acta Otorhinolaryngol Ital.

[27] Srikanth V, Maczurek A, Phan T, Steele M, Westcott B, Juskiw D, Munch G (2011). Advanced glycation endproducts and their receptor RAGE in Alzheimer's disease. Neurobiol Aging.

[28] Vicente Miranda H, Outeiro TF (2010). The sour side of neurodegenerative disorders: the effects of protein glycation. J Pathol.

[29] Wang W, Zhang LS, Zinsmaier AK, Patterson G, Leptich EJ, Shoemaker SL, Yatskievych TA, Gibboni R, Pace E, Luo H, Zhang J, Yang S, Bao S (2019). Neuroinflammation mediates noise-induced synaptic imbalance and tinnitus in rodent models. PLoS Biol.

[30] Wen TH, Afroz S, Reinhard SM, Palacios AR, Tapia K, Binder DK, Razak KA, Ethell IM (2018). Genetic reduction of matrix metalloproteinase-9 promotes formation of perineuronal nets around parvalbumin-expressing interneurons and normalizes auditory cortex responses in developing Fmr1 knock-out mice. Cereb Cortex.

[31] Xing Y, Ji Q, Li X, Ming J, Zhang N, Zha D, Lin Y (2017). Asiaticoside protects cochlear hair cells from high glucose-induced oxidative stress via suppressing AGEs/RAGE/NF-kappaB pathway. Biomed Pharmacother.

