# Supplementary material for: Overexpression of the receptor for advanced glycation end-products in the auditory cortex of rats with noise-induced hearing loss
Source: BMC Neurosci. 2021 May 21;22:38. doi: 10.1186/s12868-021-00642-3 (PMC8139161; doi:10.1186/s12868-021-00642-3)
Supplement: Supplementary file 1 — Additional file 1: Table S1. The auditory brainstem response (ABR) thresholds at pre- and post-noise exposures. [file 12868_2021_642_MOESM1_ESM.docx]

**Table S1** The auditory brainstem response (ABR) thresholds at pre- and post-noise exposures.

| Frequencies | Control group | | Noise immediate group | | Noise 4-week group | |
| --- | --- | --- | --- | --- | --- | --- |
|  | Pre (mean [SD]) | Post (mean [SD]) | Pre (mean [SD]) | Post (mean [SD]) | Pre (mean [SD]) | Post (mean [SD]) |
| 4 kHz | 31.25 (6.4) | 35.00 (7.56) | 27.5 (4.63) | 70.00 (13.09) | 35.00 (9.26) | 67.50 (11.65) |
| 8 kHz | 33.75 (7.44) | 43.75 (5.18) | 32.5 (7.07) | 75.00 (16.90) | 38.75 (6.41) | 70.00 (17.73) |
| 16 kHz | 38.75 (9.91) | 38.75 (11.26) | 31.25 (3.54) | 68.75 (11.26) | 27.50 (7.07) | 66.25 (15.06) |
| 32 kHz | 31.25 (6.41) | 36.25 (5.18) | 27.5 (4.63) | 70.00 (13.09) | 35.00 (9.26) | 67.50 (11.65) |

SD: standard deviation
