# Supplementary material for: Overexpression of the receptor for advanced glycation end-products in the auditory cortex of rats with noise-induced hearing loss
Source: BMC Neurosci. 2021 May 21;22:38. doi: 10.1186/s12868-021-00642-3 (PMC8139161; doi:10.1186/s12868-021-00642-3)

**Figure S1.** The gel images for figure 5.

A. Immediately

1. cytosolic receptor for advanced glycation end-products (RAGE) (MAB1179)


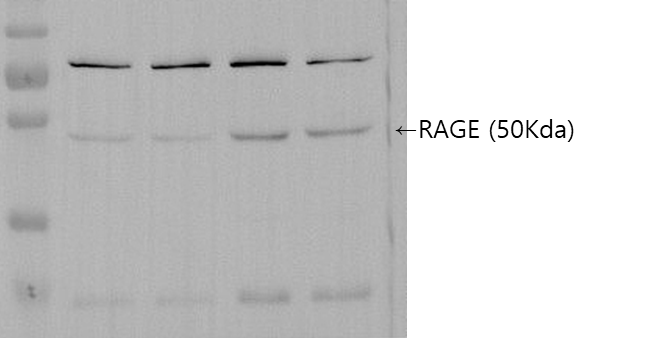


2. βActin


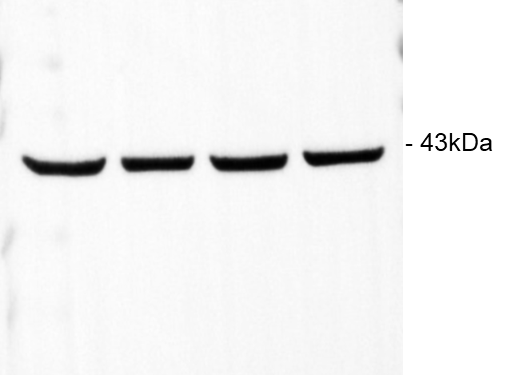


3. nuclear RAGE (Ab3611)


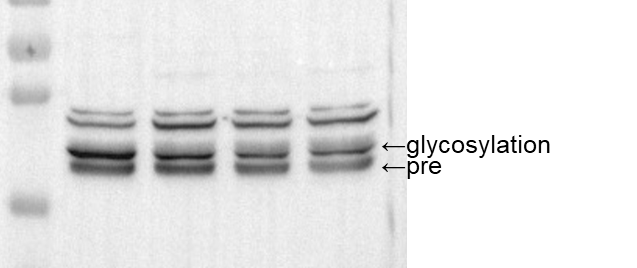


4. HDAC1


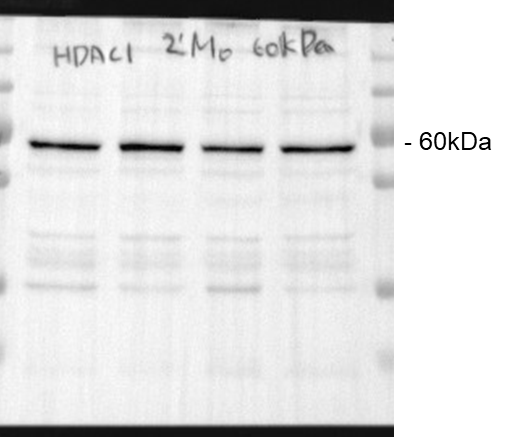


B. 4-week

1. cytosolic RAGE (MAB1179)


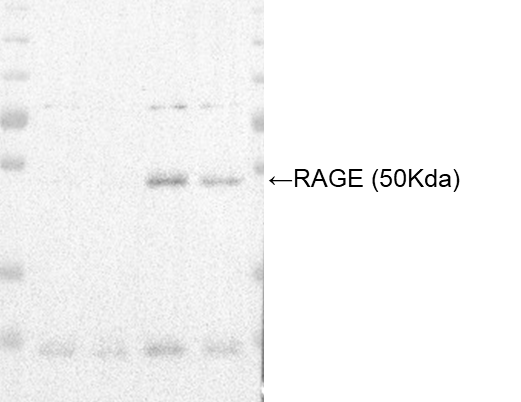


2. βActin


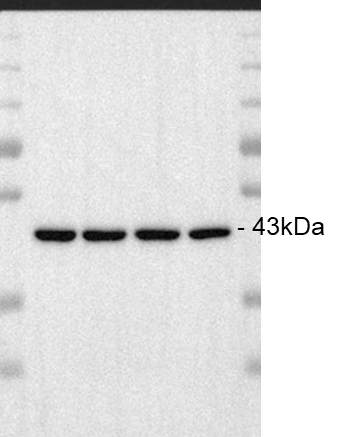


3. nuclear RAGE (ab3611)


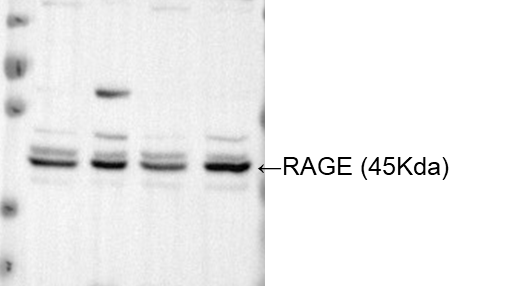


4. HDAC1


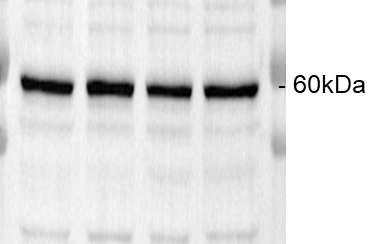


**Figure S2** The gel images for figure 6.

1. cytosolic RAGE (MAB1179)


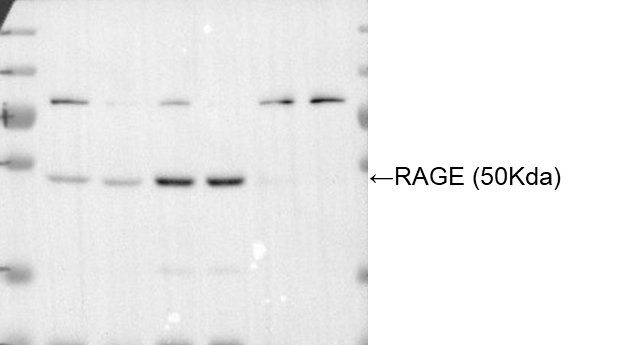


2. βActin


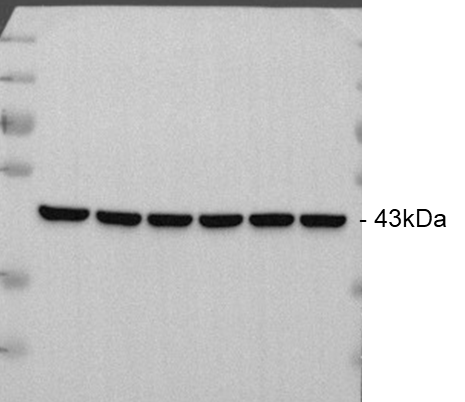


3. nuclear RAGE (ab3611)


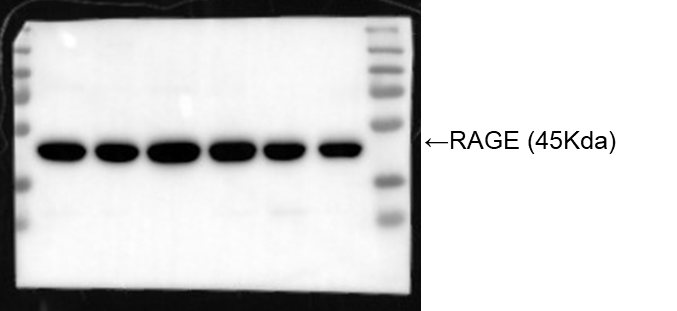


4. HDAC1


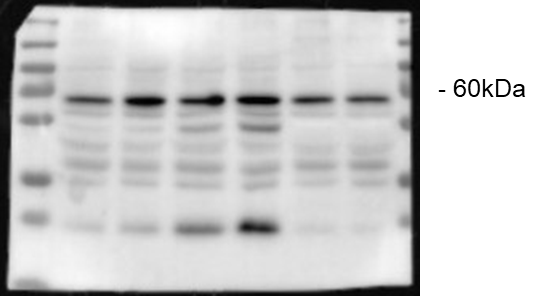


5. MMP9


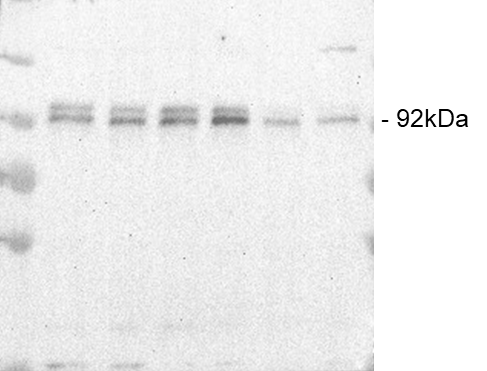

Supplement: Supplementary file 2 — Additional file 2: Figure S1. The gel images for Figure 5. Figure S2. The gel images for figure 6. [file 12868_2021_642_MOESM2_ESM.docx]
