# Supplementary material for: Overexpression of the receptor for advanced glycation end-products in the auditory cortex of rats with noise-induced hearing loss
Source: BMC Neurosci. 2021 May 21;22:38. doi: 10.1186/s12868-021-00642-3 (PMC8139161; doi:10.1186/s12868-021-00642-3)
Supplement: Supplementary file 3 — Additional file 3: Table S2. The auditory brainstem response (ABR) thresholds at pre- and post-noise exposures in control, noise, and noise+SB-3CT groups. [file 12868_2021_642_MOESM3_ESM.docx]

**Table S2** The auditory brainstem response (ABR) thresholds at pre- and post-noise exposures in control, noise, and noise+SB-3CT groups.

| Frequencies | Control group | | Noise group | | Noise+SB-3CT group | |
| --- | --- | --- | --- | --- | --- | --- |
|  | Pre (mean [SD]) | Post (mean [SD]) | Pre (mean [SD]) | Post (mean [SD]) | Pre (mean [SD]) | Post (mean [SD]) |
| 4 kHz | 26.25 (5.18) | 23.75 (7.44) | 27.5 (4.63) | 72.5 (17.53) | 35 (9.26) | 68.75 (14.58) |
| 8 kHz | 30 (0.00) | 22.5 (4.63) | 32.5 (7.07) | 80 (12.04) | 38.75 (6.41) | 73.75 (12.64) |
| 16 kHz | 28.75 (3.54) | 28.75 (11.26) | 31.25 (3.54) | 70 (14.14) | 27.5 (7.07) | 68.75 (9.59) |
| 32 kHz | 40 (0.00) | 36.25 (7.44) | 43.75 (5.18) | 76.25 (15.06) | 51.25 (30.44) | 73.75 (23.26) |

SD: standard deviation
